# Complex associations between anxiety, depression, and resilience in a college student sample: a network analysis

**DOI:** 10.3389/fpsyt.2025.1502252

**Published:** 2025-05-14

**Authors:** Hui Wang, Min Wang, Xiuchao Wang, Tingwei Feng, Xufeng Liu, Wei Xiao

**Affiliations:** ^1^ Department of Military Medical Psychology, Air Force Military Medical University, Shanxi, China; ^2^ Sleep Psychosomatic Department, Leshan Traditional Chinese Medicine Hospital, Leshan, China

**Keywords:** anxiety, depression, network analysis, resilience, bridge expected influence

## Abstract

**Background:**

Anxiety and depression have significant impacts on individuals’ mental health and social functioning, particularly among college students. Psychological resilience is considered a crucial resource for coping with adversity and stress and may play a key role in alleviating anxiety and depression symptoms. The aim of this study is to explore the finer-grained potential relationships between psychological resilience, anxiety, and depression among college students.

**Methods:**

This study employed network analysis to examine the psychological resilience, anxiety, and depression status of a randomly sampled cohort of 855 college students (51.8% female; M = 18.70, SD = 1.13). Statistical analyses and network visualization were conducted using R version 4.2.2 and the qgraph package. Bridge centrality indices of variables within the network were computed, with particular emphasis on the significance of bridge symptoms within the network structure.

**Results:**

Significant covariation was observed between anxiety and depression symptoms. Psychological resilience exhibited a negative correlation with both anxiety and depression, with a negative bridge expected influence value for R10 “Can handle unpleasant feelings”, indicating a potential protective role of psychological resilience in mitigating these mental health issues. R10 “Can handle unpleasant feelings” occupies the most central position within the psychological resilience network, with the smallest BEI value (-0.01), indicating its protective role in the overall network. To some extent, it can regulate anxiety and depression symptoms.

**Conclusion:**

This study highlights the complex interrelationships between psychological resilience, anxiety, and depression among college students through network analysis. Bridge expected influence analysis identified “R10” as a protective factor and “A7” as a key risk factor. The findings suggest that interventions targeting bridge symptoms and enhancing resilience may help alleviate anxiety and depression. Prioritizing these two symptoms in future research could yield greater intervention benefits.

## 1 Introduction

Anxiety and depression are among the most prevalent mental health disorders and frequently co-occur. Studies indicate that individuals diagnosed with anxiety often exhibit depressive symptoms, and vice versa, with comorbidity rates ranging from 40% to 60% in various populations (1–3). This high comorbidity significantly worsens disease prognosis, increases symptom severity, and complicates treatment strategies. Understanding the shared and distinct mechanisms underlying these disorders is crucial for developing more targeted interventions (4). In China, the prevalence of anxiety and depression among college students has been reported to range between 30% and 40% (5), while in North America, approximately 20% of college students report symptoms of anxiety or depression (6, 7). These findings highlight the widespread nature of these disorders in major global contexts. These findings highlight the widespread nature of these disorders and emphasize the need for further exploration of their underlying mechanisms. Anxiety and depression impose significant burdens on individuals and society. They severely affect daily functioning, academic performance, and overall well-being (8, 9). Additionally, these disorders contribute to substantial economic losses, with global productivity losses estimated at $1.15 trillion, a figure projected to triple by 2030 (10, 11). Given their widespread impact, identifying protective factors and effective interventions is essential for mitigating these consequences.

Research suggests that various predisposing factors, such as genetic vulnerability, early-life stress, and maladaptive coping mechanisms, increase the risk of anxiety and depression. Conversely, protective factors, such as strong social support, emotional regulation strategies, and resilience, help individuals cope with psychological distress (12–15).

Despite the growing body of evidence supporting the protective role of resilience, some studies have reported inconsistent findings. For example, while some research suggests that high resilience significantly reduces the risk of depression and anxiety, others indicate that resilience may only partially mediate the impact of stress on mental health outcomes (16). Furthermore, existing studies often rely on traditional variable-centered models, which may not fully capture the complex interactions between resilience, anxiety, and depression at a more granular level (17).

Resilience, as a critical concept in positive psychology, is defined as a personal resource for coping with or overcoming various adversities and perceived stress (18, 19). The definition of resilience suggests that it can reduce the risk of anxiety and depression. The study found that resilience, as an intrinsic protective factor for individuals, is significantly negatively correlated with depression and anxiety (20). For example, resilience was significantly and negatively correlated with moderate and severe anxiety: the odds for moderate and severe anxiety were significantly reduced among students who considered themselves to be of strong character. This is manifested as a one-standard deviation in-crease in resilience factors, a 76% reduction in the odds of moderate anxiety and a twofold reduction in the odds of severe anxiety (21). Some guidelines refer to exercise as recommended therapeutic strategies for depression (22). Skrove et al. found through questionnaire that resilience is an important factor in protecting adolescents from depression and anxiety in the setting of inadequate drug use and physical activity (23). The CD-RISC scale can predict positive emotions by measuring resilience (24), so resilience can predict psycho-logical disorders caused by the obstruction of positive emotions (25), such as depression, anxiety, etc. (26). Research by Bitsika et al. found that resilience can alleviate depression and anxiety from developing more severely (27, 28).

In this study, we conducted network analysis on the dimensional levels of psychological resilience, anxiety, and depression. Network analysis is an emerging data-driven method utilized for examining and visualizing the interactions between symptoms or the absence of symptoms (29). From the perspective of network theory, mental disorders arise from active interactions between symptoms or non-symptoms, rather than being solely inferred from passive deductions of underlying latent constructs. Building upon prior research (30), where dimensions of psychopathological structure are represented as nodes, network analysis elucidates the interactions between different dimensions, described as edges. Therefore, network analysis aids in investigating specific pathways linking the dimensions of psychological resilience with differences in depression and anxiety. This approach diverges from traditional latent variable models and promises to provide new in-sights into potential mechanisms for theoretical applications (31).

Network analysis is a visual model consisting of a series of nodes and edges representing interactions between nodes. In the fields of psychometrics and clinical medicine, it is commonly used to explore the interactions and underlying connections between psychological variables or symptoms. Centrality indices, also known as centrality measures, are quantitative metrics assessing the coreness of nodes within a network structure, used to understand the importance of nodes within the entire network. Recently, clinical psychologists have established network models of internal symptoms within mental disorders. Symptoms connecting two mental disorders are termed “bridge symptoms,” yet in most communities, the centrality indices and bridging nodes of these bridge symptoms have not been clearly delineated. This study focuses on designing interventions around centrality nodes of psychological resilience with anxiety and psychological resilience with depression, providing a theoretical foundation and effective targets for clinical interventions in this field.

Despite growing research on psychological resilience, anxiety, and depression, their interplay at the dimensional level remains insufficiently understood. Few studies have applied network analysis to examine these relationships, and the role of key bridge symptoms and central nodes linking resilience to anxiety and depression remains unclear. To address this gap, we constructed a network model encompassing psychological resilience, anxiety, and depression and analyzed its structural characteristics. This study aims to (1) explore the relationships between the dimensions of psychological resilience, anxiety, and depression, (2) identify pivotal central nodes, (3) examine differences in psychological well-being between rural and urban students, and (4) pinpoint key bridging nodes that facilitate the propagation of positive or negative effects across the network. By achieving these objectives, we seek to provide theoretical insights into the pathological pathways linking resilience with anxiety and depression and offer practical implications for clinical interventions.

## 2 Materials and methods

### 2.1 Participants

This study employed a cross-sectional design and was conducted from March to August 2024. A two-stage random sampling method was used to select undergraduate students from universities in Xi’an and Changsha, China. Students in Xi’an completed paper-based questionnaires offline, while those in Changsha completed the survey online via the platform (www.wjx.cn). The required sample size was calculated using the GPower software (α = 0.05, power = 0.8), yielding an estimated minimum effective sample size of 750 participants. Ultimately, 855 valid questionnaires were included in the final analysis. Inclusion criteria: (1) Full-time enrolled undergraduate students. (2) Provided informed consent to participate in the study. (3) Aged between 16 and 24 years. Exclusion criteria: (1) Questionnaires with less than 90% completion. (2) Responses exhibiting obvious patterns or inconsistencies. (3) Missing demographic information. A total of 897 questionnaires were collected, and 42 were excluded due to incompleteness, resulting in 855 valid responses (valid response rate: 95.3%). This study did not require participants to provide records of prior psychiatric diagnoses. The assessment results from the scales used reflect only current symptom levels. In future research, we aim to conduct longitudinal network analyses across different populations, comparing cross-sectional network structures at different time points and among various demographic groups. This study did not require participants to provide records of prior psychiatric diagnoses, and the scale-based assessments reflect only current symptom levels. We aim to conduct future longitudinal network analyses among different subpopulations, comparing cross-sectional networks across time points and groups. If any key variables (anxiety and depression scores, demographic variables) had more than 10% missing data, the corresponding case was excluded from the analysis.

The average (SD) age of the undergraduate individuals, ranging from 16 to 26, was 19.10 (1.42) years old. Among them, 412 male and 443 female undergraduates, 390 sole offspring and 465 non-sole off-spring, 354 urban students and 501 rural students.

The current study was reviewed and approved by the Medical Ethics Committee of the First Affiliated Hospital of the Fourth Military Medical University (No. KY20222135-C-1) (32). The study was conducted in accordance with the Declaration of Helsinki guidelines. After reading the informed consent, participants can complete the following survey if they want to further participate in this study. We will try to protect participants’ privacy.

### 2.2 Measurements

#### 2.2.1 10-item Connor–Davidson Resilience Scale (CD-RISC-10)

The CD-RISC-10 is a widely self-report questionnaire to assess resilience in different populations, including adolescents, elderly individuals and psychiatric patients (Camp-bell-Sills and Stein, 2007). Item is rated on a 5-point scale range from 0 (“not true at all”) to 4 (“true nearly all the time”), and higher total scores reflect greater ability to cope with adversity. The Chinese version was used. The internal consistency of CD-RISC-10 in the pre-sent study was excellent (α = 0.94) (33). The Chinese version of the CD-RISC-10 has been extensively validated in various populations, including university students, elderly individuals, and psychiatric patients, demonstrating good reliability and validity. Reliability: Cronbach’s α = 0.91 (original study)/0.94 (this study); test-retest reliability ICC = 0.86 (2-week interval). Confirmatory factor analysis (CFA) yielded CFI = 0.95, TLI = 0.93.

#### 2.2.2 Generalized anxiety disorder 7-Item questionnaire (GAD-7)

The GAD-7 is a valid and efficient self-report questionnaire for screening the frequency of symptoms of generalized anxiety disorder (GAD) over the last 2 weeks (A brief measure for assessing generalized anxiety disorder: the GAD-7). GAD-7 has 7 items and each item varies from 0 to 3 (point referred to “not at all”, “several days”, “more than half the days”, and “nearly every day”, respectively). The sum of scores ranges from 0 to 21, and the higher the total score, the higher the level of anxiety severity. The Chinese version was used. The internal consistency of GAD-7 in this study was excellent (α = 0.93) (34).

#### 2.2.3 PHQ-9

The Patient Health Questionnaire depression module (PHQ-9) is a 9-item self-administered instrument used for detecting depression and assessing severity of de-pression over the prior 2 weeks on a 0–3 point Likert scale (point referred to “not at all”, “several days”, “more than half the days”, and “nearly every day”, respectively). (The PHQ-9: validity of a brief depression severity measure). The sum of scores ranges from 0 to 21, and the higher the total score, the higher the level of depression severity. It is commonly used in clinical depression diagnosis and general population research studies. The Chinese version was used. The questionnaire in the present study had excellent internal consistency (α = 0.89) (35).

### 2.3 Statistical analysis

We used SPSS 22.0 software to calculate the means, standard deviations and Cronbach’s α coefficients of CD-RISC-10, GAD-7 and PHQ-9. We used R 4.3.1 software to construct the anxiety- resilience ideation and depression- resilience ideation network models and evaluate the bridge expected influence (BEI) indices of the nodes in the networks.

#### 2.3.1 Network model construction

The network was estimated by a Gaussian graphical model (GGM) with the R pack-age qgraph (36, 37). After controlling for the influences of all other symptoms in the network, this model draws out an undirected network in which edges represent the partial correlation coefficient between two symptoms (38, 39). GGM was regularized by graphical LASSO (Least Absolute Shrinkage and Selection Operator) algorithm. This statistical regularization technique can shrink all edges and make small edges become zero-weight edges to represent obtain a more stable and interpretable network. The GGM adjustment parameter was set to the recommended value of 0.5 to reach a good balance between the sensitivity and specificity of discovering true edges (40, 41). In the visual representation of the network, the red edge the negative partial correlation between nodes, whereas the blue edge rep-resent the positive partial correlation between nodes, with thicker edges indicating the stronger correlation between two nodes. We estimated the network containing resilience, anxiety and depression symptoms (i.e. R-A-D network).

The R package boonet was used to estimate the robustness of the network (42). First, we evaluated the accuracy of edge weights by computing their 95% confidence intervals (CIs) using nonparametric bootstrapping with 2000 samples. Second, the stability of node strengths and node bridge strengths were evaluated by calculating the correlation stability (CS) coefficient using a case-dropping subset bootstrapped with 2000 samples. It has been recommended that the value of the CS coefficient should not be lower than 0.25 and preferably higher than 0.50 (43). Third, we calculated bootstrapped difference tests (α = 0.05) with 2000 samples for edge weights, node strengths and node bridge strengths to assess whether two edge weights significantly differed from one another.

Studies have shown that strength is the most reliable centrality index for evaluating the importance of nodes in networks (44). Strength centrality represents the sum of all absolute values of the weights of the edges connected to a node, reflecting the possibility that the activation of a certain node may lead to the activation of others. Higher strength values indicate greater importance in the network. The strength centrality of each symptom was calculated by the R package qgraph (45, 46). In addition, we computed the bridge strength centrality of each symptom by the R package to identify bridge symptoms (47). Node bridge strength is a node’s total connectivity with other communities and is calculated as the sum of all absolute values of the weights of the edges that connect a given node to the nodes in the other communities. Higher node bridge strength values indicate a greater ex-tent of increased risk of contagion to other communities (48). In the present network, nodes were divided into two communities: one community included nine depressive symptoms (PHQ-9) and seven anxiety symptoms (GAD-7), and the other community consisted of 10-item Connor–Davidson Resilience Scale (CD-RISC-10). Bridge symptoms were identified using a blind 80th percentile cutoff on the bridge strength scores (49).

Prior to conducting network analysis, we examined key assumptions required for the Gaussian graphical model (GGM). All core variables (CD-RISC-10, GAD-7, PHQ-9) were treated as continuous and assessed for approximate normality. No extreme outliers or violations of multivariate normality were observed. Multicollinearity among items was also checked and found to be within acceptable limits. The sample size (N = 855) was sufficiently large to ensure stable and reliable estimation of network parameters, as recommended in network psychometrics literature. These checks support the appropriateness of using GGM with LASSO regularization for estimating the current symptom network.

## 3 Results

### 3.1 Descriptive statistics

The average (SD) age of the undergraduate individuals, ranging from 16 to 26, was 19.10 (1.42) years old. The participants included 412 male and 443 female undergraduates, 390 sole offspring and 465 non-sole offspring, 354 urban students and 501 rural students (as shown in **Tables 1**, **2**).

**Table 1:** The means, standard deviations and bridge expected influences of the items in the resilience -anxiety-depression ideation network.

Items	Abbreviation	M	SD	BEI	Skewness	Kurtosis
Resilience symptoms (CD-RISC-10)						
1 Able to adapt to change	R1	2.97	0.86	-0.24	-0.91	1.17
2 Can deal with whatever comes	R2	2.75	0.86	0.96	-0.65	0.75
3 Tries to see humorous side of problems	R3	2.94	0.90	0.98	-0.83	0.77
4 Coping with stress can strengthen me	R4	2.99	0.93	0.27	-0.97	1.08
5 Tend to bounce back after illness or hardship	R5	2.85	0.97	-1.07	-0.72	0.27
6 Can achieve goals despite obstacles	R6	2.93	0.92	-1.17	-0.8	0.62
7 Can stay focused under pressure	R7	2.59	0.96	-0.67	-0.33	-0.39
8 Not easily discouraged by failure	R8	3.01	0.90	-1.56	-0.93	1.02
9 Thinks of self as strong person	R9	2.97	0.94	-1.28	-0.92	0.8
10 Can handle unpleasant feelings	R10	2.95	0.92	-0.01	-0.82	0.61
Anxiety items (GAD-7)						
1 Feeling nervous, anxious, or on edge	A1	0.78	0.84	-1.39	1.11	0.92
2 Not being able to stop or control worrying	A2	0.53	0.79	1.29	1.57	2.03
3 Worrying too much about different things	A3	0.60	0.83	-0.20	1.39	1.34
4 Trouble relaxing	A4	0.47	0.80	0.61	1.79	2.62
5 Being so restless that it is hard to sit still	A5	0.32	0.69	-0.13	2.45	5.83
6 Becoming easily annoyed or irritable	A6	0.40	0.70	-0.78	1.91	3.6
7 Feeling afraid as if something awful might happen	A7	0.31	0.67	-1.55	2.52	6.31
Depression symptoms (PHQ-9)						
1 Anhedonia	D1	0.69	0.80	0.52	1.17	1.12
2 Depressed or sad mood	D2	0.54	0.75	0.08	1.47	1.98
3 Sleep difficulties	D3	0.60	0.85	-1.09	1.39	1.23
4 Fatigue	D4	0.77	0.85	0.52	1.06	0.62
5 Appetite change	D5	0.51	0.82	0.68	1.67	2.2
6 Feeling of worthlessness	D6	0.41	0.73	-0.29	1.94	3.46
7 Concentration difficulties	D7	0.50	0.77	-0.73	1.63	2.29
8 Psychomotor agitation/retardation	D8	0.25	0.58	-0.07	2.66	7.63
9 Thoughts of death	D9	0.11	0.43	-2.48	4.94	26.77

**Table 2:** There are differences in levels of psychological resilience, anxiety and depression across different demographic variables. (as shown in **Table 2**).

		N	Anxiety	Depression	Resilience
Gender	male	412	3.71 ± 4.73	4.33 ± 5.06	29.13 ± 8.28
	female	443	3.14 ± 4.10	4.47 ± 4.75	28.79 ± 6.65
	t		1.890	-0.394	0.656
	*p*		0.059	0.694	0.512
family location	urban	501	3.11 ± 3.99	4.12 ± 4.92	29.57 ± 6.94
	rura	354	3.84 ± 4.94	4.81 ± 5.48	28.08 ± 8.12
	t		-2.392	-2.031	2.811
	*p*		0.017	0.043	0.005
only child	yes	389	3.77 ± 4.69	4.44 ± 4.92	29.51 ± 7.60
	no	465	3.12 ± 4.16	4.38 ± 4.89	28.46 ± 7.33
	t		2.124	0.167	2.058
	*p*		0.034	0.867	0.040

### 3.2 Network analysis

**Figure 1:**
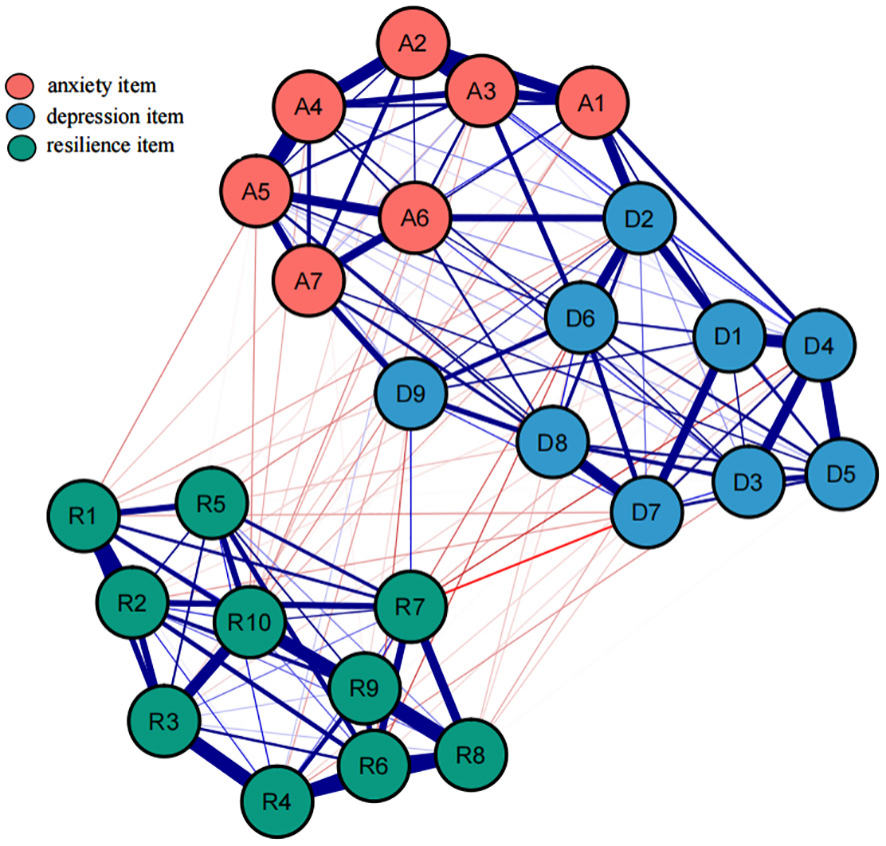
An estimated network model for resilience, anxiety and depression item in the total sample (N = 855). Blue edges represent positive correlations between the two nodes, while red edges represent negative correlations. The thickness of the edges reflects the magnitude of the correlation. The ring around the nodes depicted its predictability.

The final network of resilience, anxiety and depression is shown in **Figure 1**. Three scales represent two communities in the network. There were several obvious characteristics in this network (see **Figure 1**). First, whether it’s anxiety or depression or resilience in the internal network structure, all edges are positively correlated. Second, in general, anxiety and depression were negatively correlated with resilience. But in this network, we can see that there is a positive correlation between R7 “can stay focused under pressure” and D9 “thoughts of death”, and R4 “coping with stress can strengthen me” is positively correlated with A6 “irritable”. Third, twelve edges with the strongest regularized partial correlation existed between A4 “trouble relaxing” and A5 “restlessness” (r = 0.31), A2 “uncontrollable worry” and A3 “Worry too much” (r = 0.30), R8 “not easily discouraged by failure” and R9 “thinks of self as strong person” (r = 0.29), R3 “tries to see humorous side of problems” and R4 “coping with stress can strengthen me” (r = 0.27), R9 “thinks of self as strong person” and R10 “can handle unpleasant feelings” (r = 0.25), A2 “uncontrollable worry” and A4 “trouble relaxing” (r = 0.24), R6 “can achieve goals despite obstacles” and R8 “not easily discouraged by failure” (r = 0.23), D1 “anhedonia” and D4 “fatigue” (r = 0.23), R4 “coping with stress can strengthen me” and R6 “can achieve goals despite obstacles” (r = 0.21), D7 “concentration difficulties” and D8 “Psychomotor agitation/retardation” (r = 0.21), A1 “Nervousness or anxiety” and A2 “uncontrollable worry” (r = 0.21), D4 “fatigue” and D5 “appetite changes” (r = 0.20).

**Figure 2:**
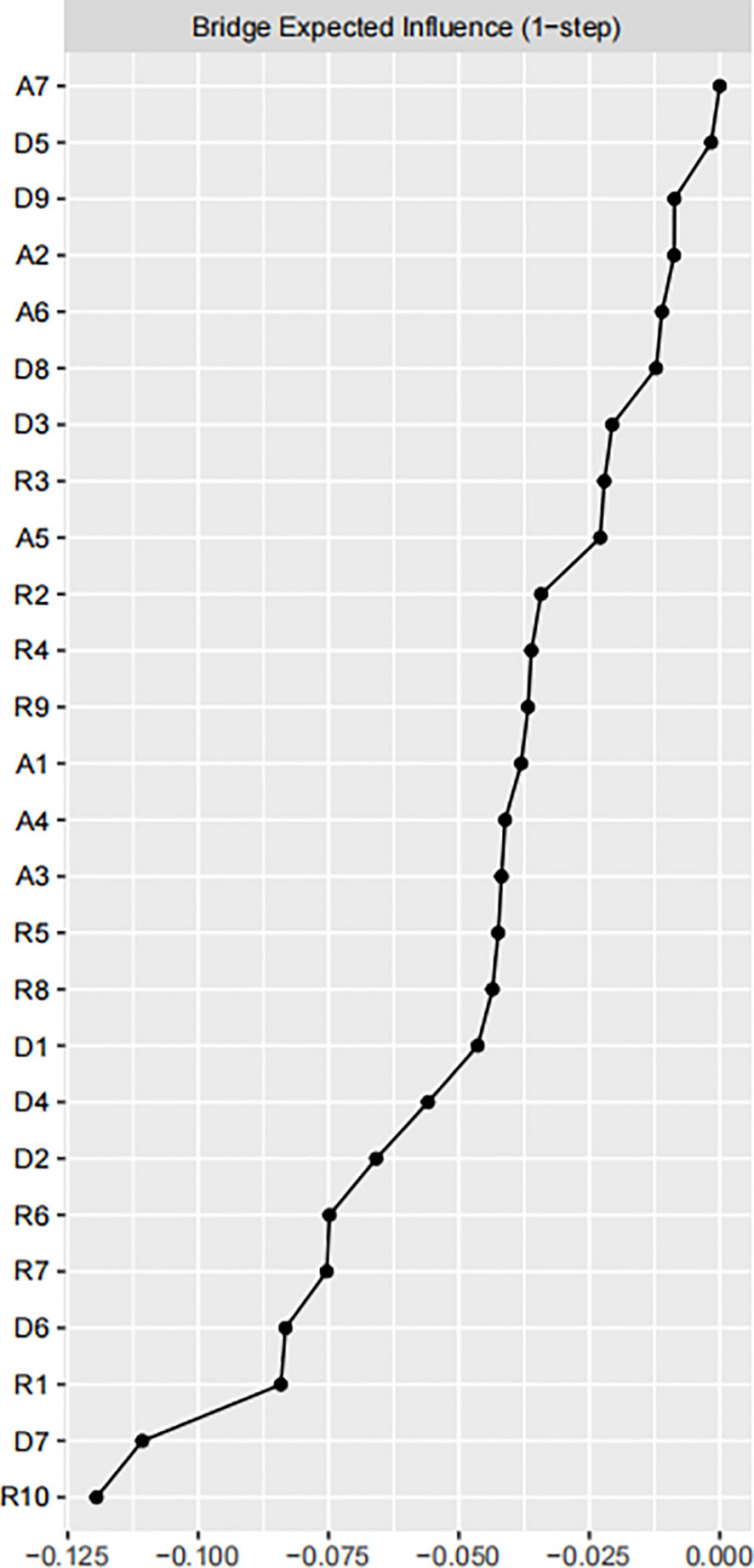
Centrality plot of the resilience, anxiety and depressive symptoms, shown as a standardized values z scores.

**Figure 3:**
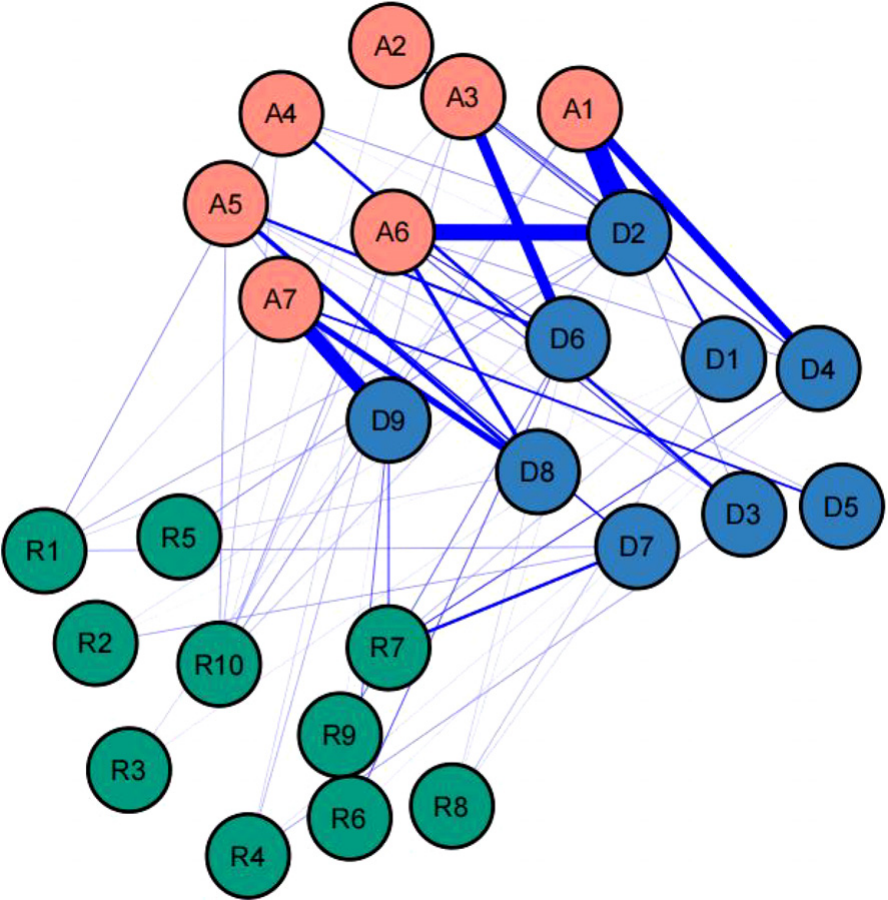
The anxiety-depression-resilience network model and the bridge expected influence.

Utilize the bootnet function of R software to estimate the stability and accuracy of the network. The narrower the CI, the more accurate the estimation of the edge weights and the centrality index (see **Supplementary Figure S1**). There are three centrality indicators of nodes, namely strength, compactness and mediation. Studies have shown that strength centrality is more stable than closeness and betweenness (45). However, when there are negatively correlated edges in the network, using the strength centrality to indicate the degree of centrality of a node in the network may distort the actual impact of the node on other nodes in the network, so the expected influence (EI) that considers both positive and negative correlated edges is used to represent node centrality (see **Figure 2**). In this network, the CS coefficient of the bridge expected influence index is 0.361, which is greater than the recommended critical value of 0.25 (see **Supplementary Figure S3**). There are some bridge symptoms in the network structure of resilience, anxiety, and depression. According to the results of bridge expected influence in **Figure 3**, the item with the highest centrality is A7, followed by D5, and R10 is the lowest. This indicates that “can handle unpleasant feelings” has the strongest ability to reduce the risk of anxiety or depression and “concentration” has the strongest ability to increase risk of contagion to anxiety and affects resilience levels. The results of bootstrapped difference test for edge weights are provided in **Supplementary Figure S2**. The results of bootstrapped difference test for node expected influences in **Supplementary Figure S4**.

## 4 Discussion

While numerous studies have investigated the relationship between anxiety or depression and resilience (50, 51). This study is the first to employ network analysis to examine the dimensional relationships between psychological resilience, anxiety, and depression within a unified framework. The findings highlight the co-occurrence of anxiety and depression symptoms and the moderating role of resilience in this relationship. Notably, key bridge symptoms were identified, serving as critical links between psychological constructs and influencing multiple mental health outcomes.

Specifically, our results indicated that urban college students have significantly lower levels of anxiety and depression and significantly higher levels of psychological resilience compared to rural college students. These findings suggest that urban students may have access to better mental health resources and support systems, which could contribute to these differences. Urban college students have significantly lower levels of anxiety and depression and significantly higher levels of psychological resilience compared to rural college students. Only children have significantly higher levels of anxiety and psychological resilience compared to non-only children.

Depending on the dynamic development of psychological resilience, external protective factors from family, school, and peer groups can enhance individual psychological resilience (52, 53).

In the general population, Tymofiyeva et al. demonstrated that resilience, as an internal protective factor, exhibits a significant negative correlation with depression and anxiety (54, 55). This finding aligns with our study results. It is noteworthy that we observed negative correlations between R7 “can stay focused under pressure” and D7 “Concentration difficulties”, R7 “can stay focused under pressure” and D4 “fatigue”, R4 “Coping with stress can strengthen me” and D3 “Sleep difficulties”, R1 “able to adapt to change” and D7 “Concentration difficulties”, R9 “thinks of self as strong person” and D6 “feeling of worthlessness”, R10 “can handle unpleasant feelings” and D1 “anhedonia”. Existing research indicates that Skrove et al, using questionnaire surveys, found that 13% of adolescents reported symptoms of depression and anxiety, with resilience being associated with lower depressive and anxiety symptoms, while inadequate drug use and physical activity were associated with higher levels of depression and anxiety. Resilience is an important factor in protect–ing adolescents from depression and anxiety in situations of inadequate drug use and physical activity. Robinson et al. (26) found that measuring resilience using the CD-RISC scale can predict positive emotions, suggesting that resilience can predict psychological disorders such as depression and anxiety caused by obstructed positive emotions (56). Therefore, through network analysis quantifying the protective or risk capacity of resilience against anxiety and depression, we calculated the bridge expected influence in the network. We found that R10 has a negative bridge expected influence value, while A7 has a positive bridge expected influence value, indicating that R10 may act as a protective factor, while A7 may manifest as a risk factor for anxiety and depression. These findings are consistent with previous research and suggest that resilience may play a buffering role in the process of individuals developing depression and anxiety.

R3, “Tries to see the humorous side of problems,” typically reflects an individual’s use of humor as a coping mechanism. While humor is generally considered a positive strategy, it can sometimes serve as a superficial or avoidant coping style, masking underlying distress rather than addressing the root causes of anxiety and depression. This might explain why R3 can act as a risk factor in certain contexts. Theoretical models suggest that avoidant coping strategies, including inappropriate humor, might fail to resolve stressors effectively, leading to an accumulation of unaddressed emotional issues and increased psychological distress.

From a clinical perspective, understanding R3 as a risk factor provides valuable insights for mental health interventions. Clinicians should be aware that while humor can be a beneficial coping tool, it might also indicate an underlying avoidance pattern in some individuals. Recognizing this dual role can help clinicians tailor their therapeutic approaches, encouraging patients to balance humor with more direct problem-solving and emotional processing techniques. This insight can inform cognitive-behavioral therapy (CBT) and other therapeutic modalities to address avoidant behaviors and foster healthier coping mechanisms. In summary, R3’ s role as a risk factor highlights the complexity of coping strategies and their varying impacts on mental health. Theoretically, it underscores the importance of distinguishing between adaptive and maladaptive coping. Clinically, it emphasizes the need for nuanced therapeutic approaches that recognize and address underlying avoidance behaviors. These insights enhance both our theoretical understanding and clinical interventions for anxiety and depression.

Our network analysis revealed strong correlations between anxiety and depression symptoms, reinforcing their co-occurrence in college students. Notably, unexpected positive correlations between resilience and anxiety/depression emerged, suggesting that resilience’s impact on mental health is context-dependent. In high-stress environments, resilience may help individuals endure adversity but could also contribute to emotional strain if coping efforts feel inadequate.

Clinically, bridge symptoms such as “handling unpleasant emotions” (protective) and “concentration difficulties” (risk factor) were identified, highlighting potential intervention targets. These findings refine our understanding of how resilience interacts with anxiety and depression and emphasize the need for tailored resilience-building and stress management strategies.

Overall, this study enhances theoretical models of resilience, anxiety, and depression by uncovering specific pathways linking these constructs. While offering insights into mental health interventions, further research is needed to validate these findings and explore underlying mechanisms.

*Limitations:* There are several limitations that should be pointed out. First, This study focused exclusively on general college students and did not include individuals with diagnosed psychiatric disorders; therefore, the applicability of the findings may not be directly generalizable to clinical populations. Second, the determination of intervention targets was based on network analysis theory, and the intervention effect of these targets on anxiety and depression still needs to be tested in practice. Although we have validated the core assumptions of the GGM (such as normality and low collinearity), it is important to note that network analysis remains sensitive to extreme values. Future studies may employ robust methods, such as Spearman correlation networks or Bayesian GGM, to further assess the stability of the results. Finally, Temperament and personality are closely related to mental disorders such as anxiety and depression, and can be further explored in future research (57, 58).

## 5 Conclusion

This study utilized network analysis to explore the relationships between psychological resilience, anxiety, and depression among college students. The findings highlight the co-occurrence of anxiety and depression symptoms and the moderating role of resilience in this relationship. Notably, A7 (“Afraid something will happen”) and D5 (“Appetite changes”) emerged as central symptoms, making them key targets for intervention and prevention strategies. These insights provide valuable guidance for developing tailored approaches to enhance resilience, reduce anxiety and depression, and promote overall mental well-being.

## Data Availability

The original contributions presented in the study are included in the article/**Supplementary Material**. Further inquiries can be directed to the corresponding authors.
